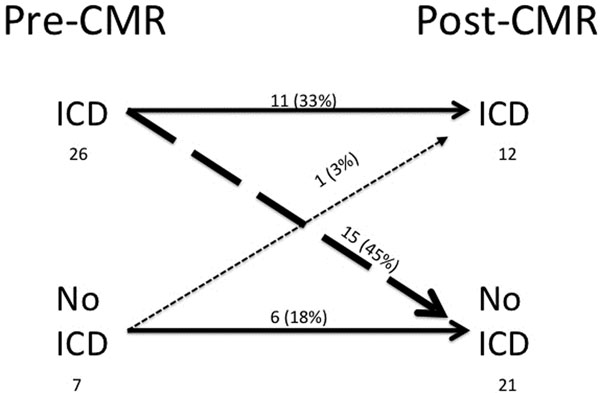# Impact of Cardiac Magnetic Resonance (CMR) on utilization of Implantable-Cardioverter-Defibrillators (ICD) for primary prophylaxis of sudden cardiac death

**DOI:** 10.1186/1532-429X-15-S1-O86

**Published:** 2013-01-30

**Authors:** Andrew Ertel, Omer Mirza, Siddique Abbasi, Vineet K Dandekar, Jaehoon Chung, Melissa Robinson Wood, Jefferson Lee, Afshin Farzaneh-Far

**Affiliations:** 1 Cardiology, University of Illinois at Chicago, Chicago, IL, USA; 2 Department of Cardiology, Brigham and Women's Hospital/Harvard Medical Center, Boston, MA, USA

## Background

Currently, left ventricular dysfunction derived from echocardiography is the main basis for determining eligibility of patients for implantable cardioverter-defibrillator (ICD) placement in the primary prevention of sudden cardiac death (SCD). However, recent trials suggest that approximately 14 to 18 patients with ventricular dysfunction need to have an ICD implanted to prevent 1 death. Given the substantial cost and potential for complications, improved risk stratification to identify patients who would benefit most from ICD implantation remains an important public health challenge. Cardiac magnetic resonance imaging (CMR) is the current gold standard for measurement of left ventricular ejection fraction (LVEF). We hypothesized that using LVEF derived from CMR compared with echocardiography may lead to a significant impact on ICD implantation rates which could translate into improved selection of patients and possible cost reductions.

## Methods

Two hundred and eleven consecutive patients referred for CMR with an echocardiographic diagnosis of cardiomyopathy and/or an LVEF of ≤45% were prospectively recruited. Of these, thirty-three patients were referred specifically to determine eligibility for ICD implantation and comprised the study population.

## Results

On the basis of CMR derived LVEF, an overall change in classification of ICD eligibility occurred in 16 patients (48%), with 1 patient (3%) reclassified to receive an ICD and 15 patients (45%) reclassified to avoid ICD implantation when they otherwise would have been implanted based on echocardiographic LVEF. In 11 patients (33%), CMR confirmed eligibility for ICD implantation as was previously suspected, and in 6 patients (18%), CMR reconfirmed that ICD implantation was not indicated. Use of CMR compared to echocardiographic LVEF led to a 43% absolute reduction in device implantation (p=0.001). None of the patients reclassified to avoid ICD implantation experienced SCD or sustained ventricular arrhythmias over a mean follow-up of 24 ±15 months.

## Conclusions

Among patients being considered for ICD implantation on the basis of prior echocardiography, use of CMR derived LVEF led to a change in ICD eligibility in 48% of cases and resulted in a 43% absolute reduction in device implantation. Moreover, none of the patients reclassified to avoid ICD implantation experienced sudden cardiac death or sustained ventricular arrhythmias over a mean follow-up of 24 ±15 months. Larger prospective studies are required to assess the safety and cost implications of these findings.

## Funding

No funding was required for this study.

**Figure 1:**